# P-1094. Systematic Approach to Increase Hand Hygiene Reliability in Underperforming High-Risk Settings

**DOI:** 10.1093/ofid/ofaf695.1289

**Published:** 2026-01-11

**Authors:** Ana M Vaughan-Malloy, Renee Moon, Jennifer Ormsby, Thomas J Sandora

**Affiliations:** Boston Children's Hospital, Boston, MA; Boston Children's Hospital, Boston, MA; Boston Children's Hospital, Boston, MA; Boston Children's Hospital, Boston, MA

## Abstract

**Background:**

Procedural and other high-risk settings have unique barriers to high reliability for hand hygiene (HH). We identified underperforming locations and required participation in local HH quality improvement (QI) initiatives.

**Methods:**

From March 2023 - November 2024, 8 high-risk settings (4 operating rooms, 3 procedural locations, and one ICU) identified champions and engaged in team-based QI to increase HH reliability to ≥ 95% for ≥ 3 consecutive months. HH Program leadership developed a toolkit, facilitated meetings, and guided teams through process improvement. Staff completed a pre-QI survey, “5 Whys” activity, and fishbone analysis and addressed identified barriers through educational campaigns, display of HH data, and other activities (Figure 1). On achieving the goal, ownership of HH efforts transitioned to local sustainability champions. HH data were collected by trained auditors in each location.

**Results:**

Initial HH reliability across the 8 settings averaged 53%. 206 pre-QI surveys were completed. 66% of respondents were clinicians, 21% technicians, and 13% other support staff. HH moments reported by staff as most challenging were after touching a patient’s surroundings (46%) and before donning (36%) or after doffing gloves (33%). The most common barriers were accessibility and functionality of HH supplies (70%) and competing priorities (35%). By October 2023 all 8 locations were actively engaged in formal QI and consistently reporting data; HH reliability averaged 81% (range, 47% to 98%). Multidisciplinary workgroups optimized supply locations and designated accountability for maintenance. An educational template adapted locally by QI groups addressed setting-specific competing demands. Six (75%) settings achieved the sustainability goal and maintained high HH reliability for ≥ 3 months; average reliability increased to 92% (range, 82% to 99%).

**Conclusion:**

A QI process facilitated by the HH Program but owned and executed at the local level led to sustained improvement in HH reliability. Common barriers across locations related to the environment, resources, and competing demands. Empowering frontline staff to engage in data review, identify opportunities, and tailor solutions was critical to drive change.

**Disclosures:**

Ana M. Vaughan-Malloy, MD, MPH, Asana: Stocks/Bonds (Public Company)|Aurora: Stocks/Bonds (Public Company)|Ayr Wellness: Stocks/Bonds (Public Company)|Bionano Genomics: Stocks/Bonds (Public Company)|Butterfly Network: Stocks/Bonds (Public Company)|Canopy Growth: Stocks/Bonds (Public Company)|Cresco Labs: Stocks/Bonds (Public Company)|CRISPR Therapeutics: Stocks/Bonds (Public Company)|Cronos Group: Stocks/Bonds (Public Company)|Curaleaf Holdings: Stocks/Bonds (Public Company)|Editas Medicine: Stocks/Bonds (Public Company)|Green Thumb Industries: Stocks/Bonds (Public Company)|High Tide: Stocks/Bonds (Public Company)|Iovance Biotherapeutics: Stocks/Bonds (Public Company)|Jushi Holdings: Stocks/Bonds (Public Company)|Moderna: Stocks/Bonds (Public Company)|Organigram Holdings: Stocks/Bonds (Public Company)|Pacific Biosciences of California: Stocks/Bonds (Public Company)|Personalis: Stocks/Bonds (Public Company)|Pfizer: Stocks/Bonds (Public Company)|SNDL: Stocks/Bonds (Public Company)|Terrascend: Stocks/Bonds (Public Company)|Tilray Brands: Stocks/Bonds (Public Company)|Trulieve: Stocks/Bonds (Public Company)

